# Multiple Disulfide-Bonded States of Native Proteins: Estimate of Number Using Probabilities of Disulfide Bond Formation

**DOI:** 10.3390/molecules25235729

**Published:** 2020-12-04

**Authors:** Philip J. Hogg

**Affiliations:** 1 The Centenary Institute, Camperdown, NSW 2050, Australia; phil.hogg@sydney.edu.au; Tel.: +61-2-8627-4716; 2 NHMRC Clinical Trials Centre, University of Sydney, Camperdown, NSW 2006, Australia

**Keywords:** disulfide bond, cystine, cysteine, probability, allosteric

## Abstract

The polypeptide backbone of proteins is held together by two main types of covalent bonds: the peptide bonds that link the amino acid residues and the disulfide bonds that link pairs of cysteine amino acids. Disulfide bonds form as a protein folds in the cell and formation was assumed to be complete when the mature protein emerges. This is not the case for some secreted human blood proteins. The blood clotting protein, fibrinogen, and the protease inhibitor, α2-macroglobulin, exist in multiple disulfide-bonded or covalent states in the circulation. Thousands of different states are predicted assuming no dependencies on disulfide bond formation. In this study, probabilities for disulfide bond formation are employed to estimate numbers of covalent states of a model polypeptide with reference to α2-macroglobulin. When disulfide formation is interdependent in a protein, the number of covalent states is greatly reduced. Theoretical estimates of the number of states will aid the conceptual and experimental challenges of investigating multiple disulfide-bonded states of a protein.

## 1. Introduction

Most disulfide bonds in mammalian proteins were acquired in vertebrate ancestors and retained as the proteins evolved [1]. Mammalian membrane and secreted proteins are particularly rich in disulfide bonds, although they are being found in proteins in all subcellular compartments. Disulfides form during maturation of proteins in the cell and bond formation has been assumed to be complete in the mature functional protein. Our recent findings indicate that this is not the case for some, perhaps many, secreted proteins [2].

Over 180,000 disulfide bonds in three-dimensional protein structures have been defined. Protein crystallization is a very exacting type of protein purification and favors the most stable, lowest energy forms of a protein. This will typically be proteins where all disulfide bonds are intact. In addition, isolating a protein that contains closely spaced cysteine thiols and preparing it for structural studies in ambient oxygen can result in oxidation of the thiols to a disulfide bond. It is not unexpected, therefore, that disulfide bonds are almost invariably intact in experimentally determined protein structures. Largely based on this information, it has been assumed that disulfide bonds are inert structural motifs that help proteins fold correctly and stabilise the tertiary and quaternary structure. I reported that two circulating blood proteins exist and function as multiple disulfide-bonded forms [2].

The redox state of the disulfide bonds in the blood clotting protein, fibrinogen, and the protease inhibitor, α2-macroglobulin, was determined using differential cysteine alkylation and mass spectrometry. The proteins were analysed in their ex vivo plasma environment and the findings represent the states of the circulating proteins. Thirteen fibrinogen disulfide bonds and twelve α2-macroglobulin disulfides are 10–50% and 10–70% reduced in the blood of healthy human donors, respectively. The functional relevance of the disulfide lability for fibrinogen conversion to fibrin polymer was explored. We observed that disulfides form upon fibrin polymerization and this is important for a functional fibrin matrix. Moreover, the covalent states of fibrinogen in plasma are influenced by physiologically relevant fluid shear forces, indicating that the different states can change in response to an external stimulus.

In this work, I have explored the question of how many disulfide-bonded forms of proteins exist. The redox state of 13 of the 17 bonds in fibrinogen and all 12 disulfides in α2-macroglobulin have been determined [2]. As we do not have a complete picture of the disulfide bonds in fibrinogen, I have focused on the results for α2-macroglobulin. Using probabilities for bond formation, the numbers of α2-macroglobulin covalent states is estimated and discussed.

## 2. Results and Discussion

α2-Macroglobulin is an abundant broad-spectrum endopeptidase inhibitor [3] produced by liver hepatocytes and macrophages that circulates in blood and functions in several biological systems [4]. Human α2-macroglobulin is a 720 kDa homo-tetrameric glycoprotein that targets proteases of the four major classes via a ‘venus flytrap’ mechanism [5]. Proteases cleave peptide bonds in the ‘bait’ region and become entrapped due to large conformational transitions in the inhibitor. These transitions expose a cysteine–glutamine thioester bond in α2-macroglobulin that is cleaved by lysine residues of proteases, resulting in their covalent attachment to the inhibitor. Proteases form 1:1 or 2:1 complex with the tetramer and remain accessible for small substrates or inhibitors. The protease-induced conformational transitions in α2-macroglobulin also expose the receptor binding domain that interacts with specific receptors on cells, leading to endocytosis of the complex and degradation in lysosomes.

The α2-macroglobulin monomer contains 11 intra-chain disulfide bonds and two inter-chain bonds involving Cys278 and Cys241 that link dimers. Seven disulfides (C48-C86, C251-C299, C269-C287, C470-C563, C595-C771, C821-C849 and C847-C883) are in the seven macroglobulin-like domains, which are antiparallel β-sandwiches comprising a three- and a four-stranded sheet. The C642-C689 disulfide is adjacent to the bait region (residues 690-728), while the C1079-C1127 bond is in the thiol-ester domain that contains the C927-Q975 thioester bond. The C921-C1321 disulfide is in the CUB domain and the receptor binding domain contains the C1352-C1467 bond.

Disulfide bonds can adopt 20 different conformations defined by the geometry of the five dihedral angles that describe the cystine residue [6]. Two of the 20 disulfide conformations, the −RHstaple and −/+RHhook bonds, are naturally strained due to stretching of the S-S bond and neighbouring α angles [6,7,8]. It is noteworthy that 5 of the 12 α2-macroglobulin disulfides have a stressed −RHstaple conformation (Table 1), suggesting that the disulfide landscape of the protein is strained. This may contribute to the general disulfide lability in the protein.

**Figure 1:**
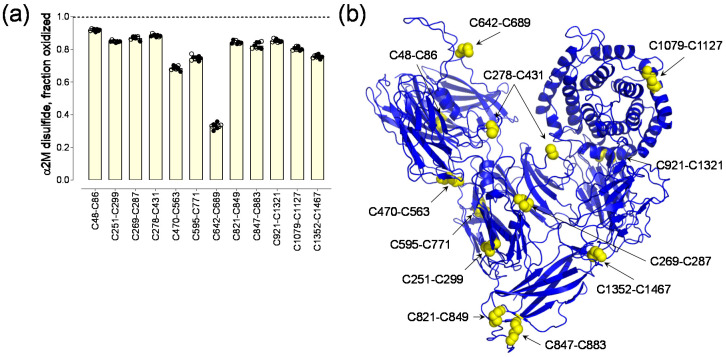
α2-Macroglobulin exists in multiple disulfide-bonded states in the circulation. (**a**) Redox states of the 12 α2-macroglobulin disulfide bonds in eight healthy human donors (5 female—solid symbols; 3 male—open symbols). The bars and errors are mean ± SD. (**b**) Ribbon structure of α2-macroglobulin monomer and the positions of the 12 disulfide bonds [5] (PDB identifier 4acq). Disulfide bond cysteines are shown as yellow spheres. The C278 and C431 residues form the inter-dimer disulfides. Figure adapted from Figure 6 of Butera and Hogg [2].

The 12 α2-macroglobulin disulfide bonds ranged from a mean of 33 to 92% formed or oxidized in eight healthy human donors (3 male, 5 female, 18–48 years old) (Figure 1) [2]. There was very little donor-to-donor variation in the redox states of the disulfides. Coefficients of variation ranged from 2.5% for the C642-C689 disulfide to 10.8% for the C847-C883 bond. A protein containing *n* disulfide bonds, where the bonds are either formed or broken, has 2*^n^* possible disulfide-bonded states. In the case of the 12 α2-macroglobulin disulfides, this equates to maximum 4096 possible disulfide-bonded states of the protein. This estimate assumes that formation of any disulfide bond in the protein is independent of any other, which is likely not the case in a protein the size and complexity of α2-macroglobulin. Using probabilities for bond formation, I estimate the theoretical numbers of α2-macroglobulin covalent states.

The mean oxidized disulfide bond fraction for the individual α2-macroglobulin disulfides represent the fraction of the bond that is formed in the population of molecules (Figure 1a). Probability (P) is defined as the extent to which an event is likely to occur, measured by the ratio of the favorable cases to the whole number of cases possible. The event for disulfides is binary, that is the bond is either formed (oxidized) or unformed (reduced) and the favorable case for this analysis is the formed bond. Approximately 5 µg of plasma α2-macroglobulin was analysed for each donor [2] that equates to ~10^12^ molecules of the protein, which is an exhaustive sampling of possible cases. Therefore, the mean oxidized fraction for the individual α2-macroglobulin disulfides represents the probability that the bond is formed in the population of molecules (Table 1). To explore the variables involved in estimating types and numbers of α2-macroglobulin states, a model polypeptide containing five disulfide bonds is simulated.

**Figure 2:**
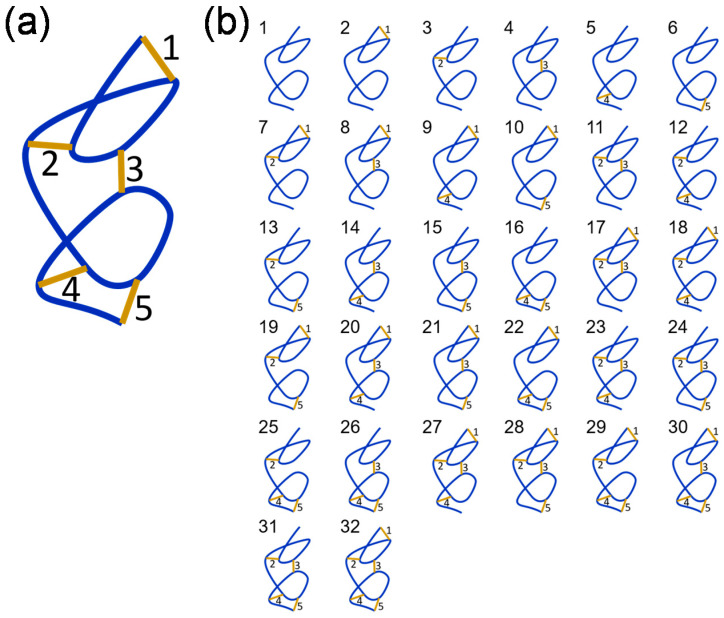
A protein containing *n* disulfide bonds has 2*^n^* possible disulfide-bonded states. (**a**) A cartoon polypeptide containing 5 disulfide bonds. (**b**) A polypeptide containing 5 disulfide bonds, where the bonds are either formed or broken, can exist in 32 possible disulfide-bonded states. Figure adapted from Supplementary Figure S3 of Butera and Hogg [2].

A polypeptide containing five disulfide bonds, where the bonds are either formed or broken, can exist in 32 (2^5^) possible disulfide-bonded states (Figure 2). These different states are represented in cartoon form in Figure 2b. It is assumed that the redox potential of the system is unchanging, so the redox states of the bonds will be static. This is a reasonable assumption for proteins that function in plasma—an environment with a stable redox potential due to small molecule and protein thiol buffering mechanisms. Listed in Table 2 are all combinations of states containing 1, 2, 3, 4 or 5 disulfide bonds, the disulfide isomeric states that contain the bond or bonds using the numbering in Figure 2b, and the probability that the states exists in the population assuming no special dependence between states. The probability is the ratio of the number of states to total number of states (32). For example, there are 10 possible states where only 2 of the 5 bonds are formed and each state occurs in 8 of the 32 possible isomers, so the probability of an isomer containing only 2 disulfide bonds is 8/32 or 0.25. For the 5 situations, that is formation of 1 bond given formation of 1, 2, 3 or 4 others, the following conditional probabilities apply:

P(1 bond) = 0.5 P(1 bond|1 other) = 0.25 P(1 bond|1 other) = 0.25 P(1 bond|2 others) = 0.125 P(1 bond|3 others) = 0.0625 P(1 bond|4 others) = 0.03125(1)

For the general case for any number of disulfides, the probability that a given number (n) of disulfide bond(s) will be formed in a protein, assuming there is no special dependence in formation of bonds, is:

P(n) = 2^−n^ (2)

For example, if a protein contains 20 disulfide bonds, the probability that all bonds are formed, assuming no special dependencies, is very small at 9.5 × 10^−7^. This analysis assumes no dependencies on formation of the bonds. This is an unlikely scenario in complex proteins containing many disulfide bonds, such as α2-macroglobulin and fibrinogen. For instance, one or more bonds might be required to form before another bond can form. These situations are illustrated using the model polypeptide and two different special conditions.

**Figure 3:**
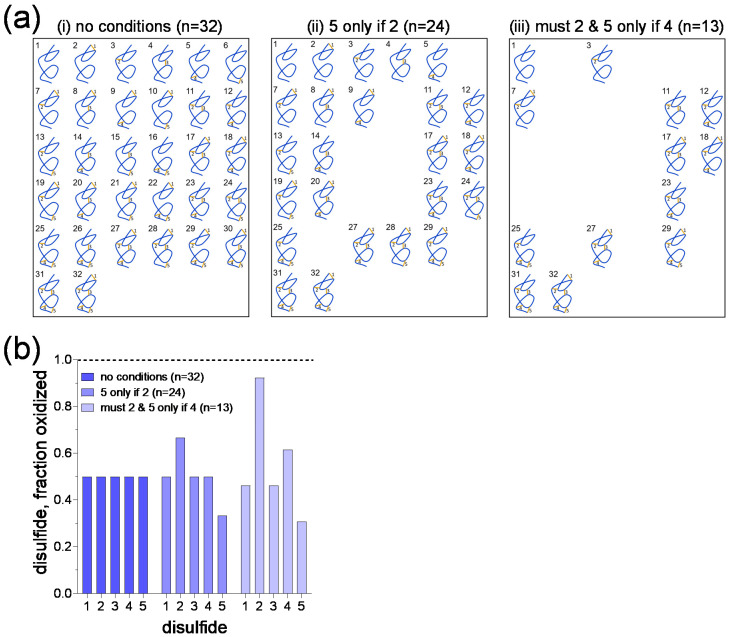
Special conditional formation of disulfide bonds. (**a**) A polypeptide containing 5 disulfide bonds, where the bonds are either formed or broken, can exist in 32 possible disulfide states (part (i)). If the condition is applied that disulfide number 5 only forms if disulfide number 2 has formed, the total number of disulfide states reduces from 32 to 24 (part (ii)). If the condition is applied that disulfide number 2 must form before any other bonds can form and disulfide number 5 forms only if disulfide number 4 has formed, the total number of disulfide states reduces further to 13 (part (iii)). (**b**) Fraction of the 5 disulfide bonds that are oxidized in the scenarios in part A, which represents the probability that the disulfide bonds are formed in the population. This representation mirrors the expression of the experimental data shown in Figure 1a.

The first condition assumes that disulfide number 5 forms only if disulfide number 2 has formed. This results in a reduction in the total number of disulfide states from 32 to 24 (Figure 3(aii)). The second condition assumes that disulfide number 2 must form before any other bonds can form and disulfide number 5 forms only if disulfide number 4 has formed. In this situation, the number of disulfide states reduces to a total of 13 (Figure 3(aiii)). The probability that each of the five disulfide bonds are formed in the different scenarios is shown in Figure 3b. It is apparent that special conditions change the probabilities for disulfide formation from a value of 0.5 for all bonds if there are no dependencies (Figure 3(ai)) to values as low as ~0.3 and high as ~0.9 if there are particular dependencies for bond formation (Figure 3(aiii)).

Note that the probability of a state containing any number of bonds is the product of the probabilities for each of the bonds:

P(a&b&c…) = P(a) × P(b) × P(c) × …(3)

This is illustrated in Table 3, which lists the probabilities for formation of only 2 or 3 bonds when there are no conditions on bond formation and for the two special cases. These calculations are applied to the 12 experimentally determined bond states in α2-macroglobulin.

From Equation (3), the probability that all 12 bonds are formed in α2-macroglobulin, assuming there is no special dependence in formation of bonds, is the product of the probabilities for each bond. For the 12 values of mean oxidized state (Table 1), this equates to a probability of 0.037. In other words, just 3.7% of circulating plasma α2-macroglobulin is predicted to be fully oxidized. If disulfide bond formation in α2-macroglobulin has special conditions, however, the total number of disulfide isomeric states will be reduced, and the incidence of fully oxidized protein will increase. On the other hand, this analysis assumes that a fully oxidized protein can indeed exist, which has not been demonstrated experimentally. It is possible, for instance, that one bond will not form if one or more other bonds have formed and so a fully oxidized protein cannot exist.

For the 12 α2-macroglobulin disulfides, 8 have probabilities of formation > 0.8 while 4 of the 12 have probabilities of formation from 0.33 to 0.76 (Figure 1a, Table 1). This indicates that the majority of the α2-macroglobulin disulfides are favored to form. The total number of α2-macroglobulin states, therefore, is much smaller than the 4096 predicted assuming no dependencies on disulfide bond formation. Even so, a hundred-fold fewer states for instance would mean that the protein circulates in ~40 different covalent forms, which is a potential complexity of protein function not envisaged for a single fully oxidized state. An example of this complexity could be the fate of the C642-C689 disulfide.

**Figure 4:**
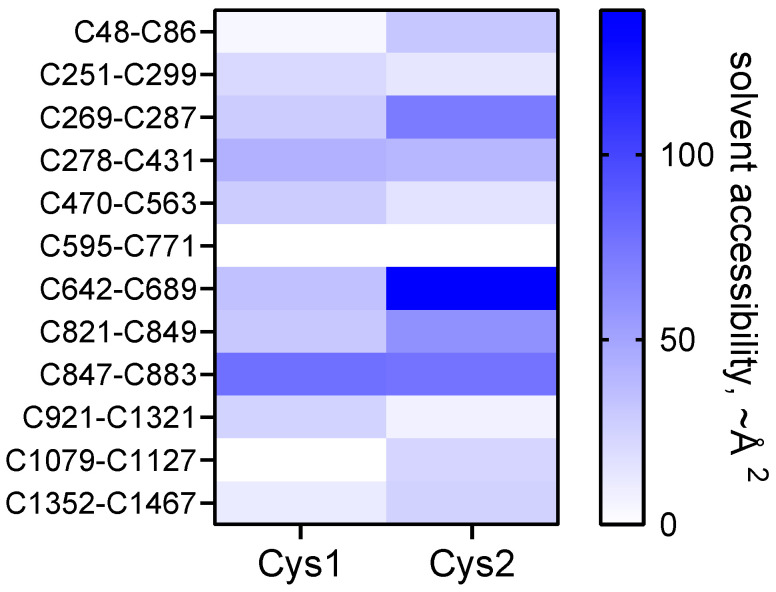
Heat map of the solvent accessibility of the 24 α2-macroglobulin disulfide bond cysteines in the homo-tetramer. The scale is the solvent accessibility in ~Å^2^ derived from DSSP [12] using the PDB identifier 4acq structure of α2-macroglobulin [5].

The C642-C689 bond adjacent to the residue 690-728 bait region is the most reduced bond in the protein, with only 1 in 3 molecules containing an oxidized bond (Table 1). This suggests that the C642-C689 disulfide might be an allosteric bond, which is a different type of disulfide bond to that described herein. Allosteric disulfide bonds are cleaved in the mature protein and control function through conformation changes as a result of bond cleavage [6,7,10]. These bonds are found on or near the protein’s surface so they are accessible to factors, such as the oxidoreductases, that manipulate their redox state [11]. The C689 residue of the C642-C689 bond is the only one of the 24 disulfide cysteines that is freely accessible to solvent in the crystal structure of the α2-macroglobulin homo-tetramer (Figure 4). It will be of interest to determine if cleavage of the C642-C689 bond by a vascular thiol isomerase [11] controls protease cleavage of the bait region. The other 11 disulfide bonds in mature α2-macroglobulin are predicted to be inaccessible to oxidoreductases, which implies their redox state is set during folding and maturation, as is the case for most of the disulfide bonds in fibrinogen [2]. It is also possible that redox state of the C642-C689 bond is controlled by formation of one or more other bonds. For instance, the probability that both the C642-C689 and nearby C48-C86 bonds are both formed in the population of molecules is 0.30 (0.33 × 0.92). It may be, for example, that both disulfides are required to be reduced or oxidised to inhibit certain proteases.

## 3. Conclusions

The conceptual and experimental challenges of dealing with many disulfide-bonded forms of a protein will be aided by theoretical estimates of the number of forms. This information will help investigators better define hypotheses and experimental strategies for tackling research questions. Future studies will refine the number and type of covalent states and explore how the different states contribute to protein function. The probabilities for bond formation approach outlined in this study is a starting point for estimating the number of disulfide-bonded forms of a protein.

## Figures and Tables

**Table 1:** Conformation of the α2-macroglobulin disulfide bonds and fraction of the bonds that are oxidized in protein populations from healthy human donors. The disulfide conformations were determined using the Disulfide Bond Analysis tool [9] and PDB identifier 4acq structure of α2-macroglobulin homo-tetramer [5]. Two of the disulfides have different conformations in 1 of the 4 molecules of the tetramer. The fraction oxidized are the mean values of eight donors (data from Figure 1a) and are equivalent to the probability that the disulfide bond is formed in the population of α2-macroglobulin molecules.

α2-Macroglobulin Disulfide	Conformation	Fraction Oxidized
C48-C86	−RHstaple	0.92
C251-C299	−LHhook	0.85
C269-C287	−RHstaple	0.87
C278-C431	−RHstaple	0.89
C470-C563	−RHspiral	0.69
C595-C771	−RHstaple	0.75
C642-C689	−LHstaple	0.33
C821-C849	−LHstaple	0.84
C847-C883	+/−LHhook	0.82
C921-C1321	−RHstaple	0.85
C1079-C1127	−RHspiral	0.81
C1352-C1467	+/−LHstaple	0.76

The C278-C431 inter-chain disulfide linking the C and D chains of the homo-tetramer has a −LHspiral conformation. The C642-C689 intra-chain disulfide in the D chain of the homo-tetramer has a +/−LHstaple conformation.

**Table 2:** A polypeptide containing 5 disulfide bonds, where the bonds are either formed or broken, can exist in 32 possible disulfide states. Shown are all combinations of states containing only 1, 2, 3, 4 or 5 disulfide bonds, the disulfide isomers that contain the bond or bonds using the numbering in Figure 2b, and the probability that the disulfide bond or bonds exist in the population assuming no dependencies between states.

Disulfides Formed	Disulfide Isomers Containing the Bond(s)	Probability
1	2,7,8,9,10,17,18,19,20,21,22,27,28,29,30,32	0.5
2	3,7,11,12,13,17,18,19,23,24,25,27,28,29,31,32	0.5
3	4,8,11,14,15,17,20,21,23,24,26,27,28,30,31,32	0.5
4	5,9,12,14,16,18,20,22,23,25,26,27,29,30,31,32	0.5
5	6,10,13,15,16,19,21,22,24,25,26,28,29,30,31,32	0.5
1,2	7,17,18,19,27,28,29,32	0.25
1,3	8,17,20,21,27,28,30,32	0.25
1,4	9,18,20,22,27,29,30,32	0.25
1,5	10,19,21,22,28,29,30,32	0.25
2,3	11,17,23,24,27,28,31,32	0.25
2,4	12,18,23,25,27,29,31,32	0.25
2,5	13,19,24,25,28,29,31,32	0.25
3,4	14,20,23,26,27,30,31,32	0.25
3,5	15,21,24,26,28,30,31,32	0.25
4,5	16,22,25,26,29,30,31,32	0.25
1,2,3	17,27,28,32	0.125
1,2,4	18,27,29,32	0.125
1,2,5	19,28,29,32	0.125
1,3,4	20,27,30,32	0.125
1,3,5	21,28,30,32	0.125
1,4,5	22,29,30,32	0.125
2,3,4	23,27,31,32	0.125
2,3,5	24,28,31,32	0.125
2,4,5	25,29,31,32	0.125
3,4,5	26,30,31,32	0.125
1,2,3,4	27,32	0.0625
1,2,3,5	28,32	0.0625
1,2,4,5	29,32	0.0625
1,3,4,5	30,32	0.0625
2,3,4,5	31,32	0.0625
1,2,3,4,5	32	0.03125

**Table 3:** Probabilities for formation of only 2 or 3 bonds in a polypeptide containing 5 disulfide bonds when there are no conditions on bond formation, and for two special cases. The disulfide isomers that contain the 2 or 3 bonds uses the numbering in Figure 2b.

Disulfide	Probability	Disulfides Formed	Disulfide Isomers	Probability *
No conditions				
1	0.50	1,3	8,17,20,21,27,28,30,32	0.25
2	0.50	1,3,5	21,28,30,32	0.125
3	0.50			
4	0.50			
5	0.50			
Bond number 5 only forms if bond number 2 has formed				
1	0.50	1,3	8,17,20,27,28,32	0.25
2	0.67	1,3,5	28,32	0.083
3	0.50			
4	0.50			
5	0.33			
Bond number 2 must form and bond number 5 forms only if bond number 4 is formed				
1	0.46	1,3	17,27,32	0.213
2	0.92	1,3,5	32	0.066
3	0.46			
4	0.62			
5	0.31			

* Probability of a state containing bonds 1 and 3 or 1, 3 and 5 is the product of the probabilities for each bond for the specific condition (Equation (3)).
